# Annotations

**Published:** 1911-08-26

**Authors:** 


### Annotations.

### An Impudent Circular.

A correspondent has forwarded to this office a manuscript letter which he has received from a midwife in Paris with whom he is totally unacquainted. The letter invites the addressee to recommend to his patients the sender's maternity home in Paris. If that were all, it might pass merely for a misguided form of advertisement, since a respectable medical man in England is little likely to send abroad his midwifery cases. There are, however, two sentences which give a veiled hint that this may be a less legitimate form of enterprise. "Ladies are taken care of," says the writer of the letter, "from beginning to end, and at every term of their pregnancy. They may be assured of the greatest discretion." If the meaning we read into these lines is not the right one, we apologise beforehand; but, to put it plainly, we conceive this to be a barefaced invitation to send patients to Paris for the purpose of having abortion procured. If this interpretation be the correct one, we think the doctor to whom the letter was sent would be doing a public service by forwarding the letter to the Commissioner of Police or the Postmaster-General. It is unfortunately true that neither of these officials can touch the midwife as long as she stays in France; but such measures as have already been taken with the Antwerp bookmakers might be possible in this case also. If any other of our readers have received similar letters, we should be glad to hear of it.

### The "Hapsburg Face."

A little attention has been attracted by a paper read by Mr. Rushton at the annual conference of the British Dental Association, in which the historical protruding Hapsburg lower lip is instanced as an example of hereditary abnormality of facial contour. The Spanish Court dentist, Dr. Aguilar, who was present, stated that the peculiarity could be traced as far back as the Emperor Maximilian I., born, by the way, in 1459. But in Dr. Galippe's study of this subject, a beautifully illustrated and interesting work upon which Mr. Rushton seems to have drawn for some of his facts, it is stated that a portrait of the German Emperor Rudolph I. (1218-1291) shows the characteristic "prognathisme inférieur." Dr. Galippe is not so optimistic as his latest exegete: he considers the facial trait a distinct stigma of degeneracy. At all events, one cannot agree with the argument put forward that intellectual ability is a proof of the absence of degeneracy, since now that Lombroso has popularised it, everybody knows of the theory of Moreau of Tours that it is just those individuals with a vein of genius in their composition who do show moral or physical degeneracy. Nor, with such a lengthy history of transmission, would one be quite prepared to agree that an adenoid curette freely used is going to be the means of razing that ancient institution, the "Hapsburg lip." What is really wanted, it seems to us, is a new and comprehensive definition of degeneracy, a term probably used more loosely to-day than any other pseudo-scientific catchword.

### "Lead Chloroform Poisoning."

A particularly interesting example of the ease with which even the most careful of newspaper editors may be misled in medical matters has occurred in an account of an inquest held at the West Ham Coroner's Court. From the report it appears that a lad died after an operation for which chloroform was administered. The readers of the journal in question are given to understand that the medical evidence attributed the cause of death to "lead chloroform poisoning." Seeing that the boy's occupation was that of a printer's assistant and he was therefore liable to be affected by lead poisoning, it becomes perfectly excusable if the lay reader gains the impression that such a condition as "lead chloroform poisoning" is a recognised risk associated with dealings in leaden type. Indeed, we may venture to surmise that at a first glance even a medical man might miss the exact point where the error lies concealed. It is, of course, in the word "lead." This, we take it, is a false transcription of the word "delayed"—an error not difficult to appreciate when the word is uttered none too distinctly or imperfectly caught, as may well happen in the environment of a coroner's court. That the death was due to delayed chloroform poisoning—unfortunately a well-known fatal sequence in general anæsthesia—is confirmed by reconsidering the report, and by the fact that the age of the lad was only fourteen years.

### The Regimentation of the Poor.

Not only in Palermo is there to be encountered strenuous opposition to the efforts of sanitarians. We learn that at our own doors the emissaries of the public health authorities are having great difficulties to contend with. The Medical Officer of Health for Willesden complains of the intense reluctance of some parents to dissociate vermin from their children's heads. Not long ago we seem to have heard of a small riot outside a school in that district, during which the school nurse incurred a fair share of practical resentment as the result of her well-meant endeavours to render certain children less accommodating as hosts to the prolific *pediculi capitis*. This resistance of the populace to the ever-extending principle of regimentation in matters of health must be truly saddening to health committees. It is now surely time that the poor should have become resigned to the invasion of their homes by innumerable inspectors; otherwise what becomes of the advantages of being poor? Still, invasion or no invasion, certain elementary principles of hygiene must be enforced. By all means amend the Children's Act to allow for the compulsory cleansing not only of the school child, but also of the other members of the family, too, and of the whole house itself. The people will not rebel, perhaps, against the ubiquitous inspector—they never yet have rebelled in this country against him.

